# Inverse Correlation Between Nesfatin-1 and Ghrelin O-Acyltransferase (GOAT) in Adolescents with Epilepsy: A Cross-Sectional Study

**DOI:** 10.3390/biom16050658

**Published:** 2026-04-28

**Authors:** Anna Sojka, Ozgun Yetkin, Yasmin Bartosik, Barbara Steinborn, Barbara Dorocka-Bobkowska, Marcin Zarowski

**Affiliations:** 1 Department of Prosthodontics and Gerostomatology, Poznań University of Medical Sciences, 60-812 Poznań, Poland; annasojka@ump.edu.pl (A.S.);; 2 Department of Developmental Neurology, Poznań University of Medical Sciences, 60-355 Poznań, Poland; 3 Doctoral School, Poznań University of Medical Sciences, 60-812 Poznań, Poland

**Keywords:** epilepsy, nesfatin-1, ghrelin o-acyltransferase, ghrelin, biomarkers, neuropeptides, adolescents

## Abstract

Nesfatin-1 and ghrelin O-acyltransferase (GOAT) have established roles in metabolic regulation and neuronal excitability, yet their relationship in epilepsy remains conflicted. This cross-sectional study compared 22 adolescent epilepsy patients (13.1 ± 2.0 years; 11 women/11 men) with 20 age-matched healthy controls (HCs) (12.3 ± 2.2 years; 8 women/12 men). Serum and salivary nesfatin-1 and GOAT levels were measured by enzyme-linked immunosorbent assay; Spearman’s rank correlation assessed inter-neuropeptide relationships. Serum nesfatin-1 was markedly elevated in patients with epilepsy compared to HCs (44.04 (interquartile range 38.19–76.72) vs. 8.65 (interquartile range 7.82–9.01) ng/mL, *p* < 0.001; ~5-fold). Serum GOAT was similarly elevated (4.90 (interquartile range 4.17–6.66) vs. 1.41 (interquartile range 1.21–1.79) ng/mL, *p* < 0.001; 3.5-fold). A significant inverse correlation between serum nesfatin-1 and GOAT levels was identified in patients with epilepsy (rho = −0.68, 95% CI [−0.86, −0.36], *p* < 0.001) but not in HCs (*p* = 0.53) and remained independent of age, sex, body mass index and epilepsy type. This inverse correlation was significant in women (rho = −0.68, *p* = 0.021) with a similar trend in men (rho = −0.53, *p* = 0.096). Salivary nesfatin-1 mirrored serum patterns (2.3-fold increase; *p* < 0.001), while salivary GOAT showed a 9-fold reduction (*p* < 0.001). This study provides the first evidence of an inverse nesfatin-1/GOAT correlation in adolescent epilepsy, suggesting disease-specific neuroendocrine dysregulation. These exploratory findings support the potential of these neuropeptides as candidate biomarkers warranting further validation and offer new insights into the metabolic-excitability axis in epilepsy.

## 1. Introduction

Epilepsy affects approximately 50 million people worldwide, making it one of the most prevalent chronic neurological disorders [1]. Despite therapeutic advances, nearly 30% of patients remain drug-resistant, creating an urgent need for reliable biomarkers that can aid diagnosis, treatment prediction, and guide personalized therapy [2,3,4]. Minimally invasive biomarkers, such as neuropeptides, could transform early diagnosis by enabling bidirectional communication between peripheral organs and the central nervous system (CNS) and enabling more targeted interventions [5,6,7].

The nesfatin-1 and ghrelin O-acyltransferase (GOAT) axis is a promising candidate due to its roles in metabolic regulation and neuronal excitability [8,9]. One crucial feature of nesfatin-1 is its ability to hyperpolarize dopaminergic neurons in the substantia nigra, suggesting it may modulate excitability in regions critical for seizure activity [10]. Similarly, ghrelin receptors are found in brain regions involved in seizure generation and propagation, such as the hippocampus, amygdala, and cortex [11]. However, the biological activity of ghrelin depends on its post-translational acylation, catalyzed exclusively by the enzyme GOAT, also known as membrane-bound O-acyltransferase domain-containing protein 4 (MBOAT4). Because GOAT serves as the “master switch” for the ghrelin system, its levels provide a direct window into the metabolic and neuronal regulation that are often disrupted in epilepsy [12]. Both peptides can cross the blood–brain barrier, enabling bidirectional communication between peripheral organs and the CNS [11,13,14].

Our recent systematic review revealed considerable variability in ghrelin findings: 37.1% reported decreased levels, 29.6% reported increased levels, and 33.3% reported no change. In contrast, nesfatin-1 demonstrated more consistent patterns, with 80% of studies reporting elevated levels in patients, particularly those who were untreated or experiencing active seizures. Higher nesfatin-1 levels correlated with seizure frequency and poorer prognosis in status epilepticus, while lower levels were observed in drug-resistant temporal lobe epilepsy [15].

We conducted this cross-sectional study with two primary objectives: first, to compare serum and salivary levels of nesfatin-1 and GOAT between adolescents with epilepsy and age-matched healthy controls (HCs); and second, to examine the relationship between these neuropeptides. We hypothesized that nesfatin-1 and GOAT would show an inverse relationship, independent of comorbid conditions, supporting their potential as epilepsy-specific biomarkers.

## 2. Materials and Methods

### 2.1. Study Design and Ethical Approval

This cross-sectional study was conducted at the Department of Developmental Neurology, Poznań University of Medical Sciences, Poznań, Poland, from November 2017 to December 2020. The study protocol was approved by the Bioethics Committee of Poznań University of Medical Sciences (approval number 420/17) and adhered to the Declaration of Helsinki. Written informed consent was obtained from all participants and their legal guardians after a comprehensive explanation of the study procedures. Participation was voluntary, and anonymity and confidentiality were maintained throughout. All procedures were designed to minimize potential burdens or risks to participants.

### 2.2. Study Population

#### 2.2.1. Epilepsy Group

Among 500 patients screened, 22 with a confirmed epilepsy diagnosis according to the International League Against Epilepsy (ILAE) were included. Exclusion criteria included: history of other neurological or psychiatric disorders, use of psychiatric medications, severe medical conditions or psychotic disorders, body mass index (BMI) ≥ 27 kg/m^2^, chronic inflammatory, malignant or gastrointestinal disorders. All participants underwent a comprehensive neurological examination by certified neurologists (M.Z. and B.S.), including detailed medical history and neurological assessment.

#### 2.2.2. Control Group

20 age-matched healthy adolescents from the same geographic area with no history of epilepsy or neurological disorders, no seizures or loss of consciousness episodes, and general good health were included. Exclusion criteria matched those of the epilepsy group.

### 2.3. Sample Collection

Biological samples were collected between 8:00 and 9:00 AM after an overnight fast (minimum 8 h). Participants refrained from eating, drinking (except water), and performing oral hygiene procedures for at least 1 h before collection.

Fasting venous blood (5 mL) was collected from the antecubital vein using the standard venipuncture technique. Blood was allowed to clot at room temperature for 30 min, then centrifuged at 3000× *g* for 5 min. Serum was immediately aliquoted and stored at −20 °C.

Unstimulated whole saliva (2 mL) was collected using the passive drooling method. Participants were instructed to allow saliva to accumulate naturally on the mouth floor and to drool into sterile collection tubes for approximately 5 min. Saliva samples underwent double centrifugation (3046.4× *g* for 10 min, repeated twice). The final supernatant was aliquoted into 0.5 mL tubes and stored at −20 °C until analysis.

### 2.4. Biochemical Analysis

Serum and salivary concentrations were measured using commercial enzyme-linked immunosorbent assay (ELISA) kits:

Nesfatin-1: Human NEFA/Nesfatin-1 ELISA Kit (Cat# E8726h; Wuhan EIAab Science Inc., Wuhan, China). Detection range: 0.312–20 ng/mL; Sensitivity: <0.12 ng/mL; Intra-assay coefficient of variation (CV): ≤6.2%; Inter-assay CV: ≤8.5%; Spike average recovery: 94%. Samples with concentrations exceeding the upper detection limit of the nesfatin-1 ELISA kit (20 ng/mL) were diluted tenfold in assay buffer and re-assayed. Back-calculated concentrations were obtained by multiplying the measured value by the dilution factor (×10). All reported nesfatin-1 values correspond to back-calculated concentrations confirmed within the linear range of the standard curve.

GOAT: Human MBOAT4/GOAT ELISA Kit (Cat# E3525h; Wuhan EIAab Science Inc., Wuhan, China). Detection range: 0.312–20 ng/mL; Sensitivity: <0.1 ng/mL; Intra-assay CV: ≤5.9%; Inter-assay CV: ≤10.1%; Spike average recovery: 102%.

All samples were analyzed in duplicate, and median values were used for statistical analysis. Samples were thawed only once immediately before analysis to prevent freeze–thaw cycles that could affect peptide stability.

### 2.5. Clinical Assessment

Anthropometric measurements were obtained using standardized procedures. Height was measured to the nearest 0.1 cm using a wall-mounted stadiometer, and weight was measured to the nearest 0.1 kg using a calibrated electronic scale. BMI was calculated as weight in kilograms divided by height in meters squared (kg/m^2^).

### 2.6. Statistical Analysis

Statistical analyses were performed using R version 4.5.2 [16]. Continuous variables were assessed for normality using the Shapiro–Wilk test. The Welch Two-Sample *t*-test was used to compare age and BMI between groups, accounting for potentially unequal variances. Although only serum GOAT levels met normality criteria in both groups, the Mann–Whitney U test was used consistently across all between-group biomarker comparisons because all three (serum/saliva Nesfatin-1, and saliva GOAT) distributions were non-normal. The relationship between nesfatin-1, GOAT, and clinical parameters was evaluated using Spearman’s rank correlation coefficient (rho). Comprehensive pairwise correlations were computed for all biomarker combinations across serum and saliva, separately for patients, controls, and the pooled sample. To assess the robustness of the primary correlation, 95% confidence intervals were derived using Fisher’s z-transformation, partial Spearman correlations controlling for age, sex, and BMI were computed using the ppcor package (version 1.1), and influence diagnostics (Cook’s distance) were performed on rank-transformed data. A non-parametric bootstrap (2000 replicates) provided additional confidence interval estimation.

### 2.7. Sample Size Considerations

Sample size was determined based on previous literature reporting nesfatin-1 and GOAT alterations in neurological disorders. Given the large effect sizes (Cohen’s d > 2.0 or equivalent non-parametric effect sizes) observed for the primary outcome measures—specifically the differences in serum nesfatin-1 and GOAT levels between epilepsy patients and controls—a sample of 20–25 participants per group provides adequate power (>0.80) to detect significant differences at α = 0.05. Nonetheless, findings should be considered exploratory and hypothesis-generating given the limited sample size.

## 3. Results

### 3.1. Demographic and Clinical Characteristics

A total of 22 adolescent patients with epilepsy and 20 age-matched HCs participated in this study (Table 1). The groups were comparable in age (epilepsy: 13.1 ± 2.0 years vs. controls: 12.3 ± 2.2 years, *p* = 0.23) and sex distribution (11 women/11 men vs. 8 women/12 men, *p* = 0.753) or BMI (epilepsy 20.01 (IQR 18.66–23.74) vs. HCs 19.82 (IQR 18.00–22.01) kg/m^2^; *p* = 0.583).

The study cohort comprised adolescents aged 11–16 years, spanning various stages of pubertal development. Biomarker alterations were observed consistently across the age range, with no significant correlation between age and nesfatin-1 (rho = 0.40, *p* = 0.056) or GOAT levels (rho = −0.39, *p* = 0.066).

The epilepsy cohort was clinically heterogeneous, including 16 patients (72.7%) with generalized epilepsy (GE) and 6 patients (27.3%) with focal epilepsy (FE). Among GE patients, 14 had idiopathic GE (IGE) and 2 had juvenile myoclonic epilepsy (JME). FE patients included 2 with FE, 2 with drug-resistant focal epilepsy, 1 with sleep-related hypermotor epilepsy (SHE), and 1 with Landau–Kleffner syndrome. All epilepsy patients were receiving anti-seizure medication (ASM) and had documented seizure activity within the three months before enrollment.

### 3.2. Serum and Salivary Biomarker Levels

**Figure 1:**
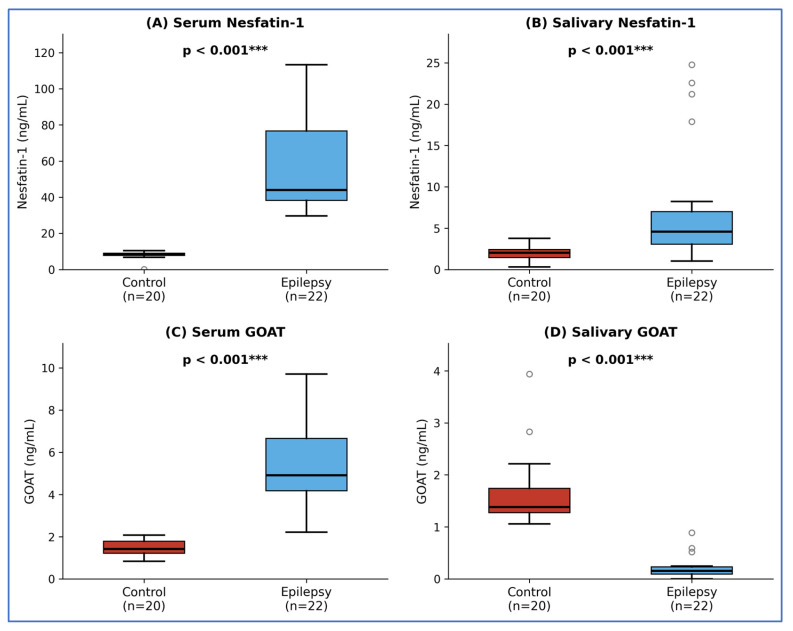
(**A**) Serum nesfatin-1; (**B**) Salivary nesfatin-1; (**C**) Serum GOAT; (**D**) Salivary GOAT. All between-group comparisons *p* < 0.001, Mann–Whitney U test. A significant sex difference was observed in serum GOAT levels (women: 5.80 (IQR 4.78–7.73) ng/mL vs. men: 4.16 (IQR 3.18–4.90) ng/mL, *p* = 0.019). No significant sex difference was observed in serum nesfatin-1, salivary nesfatin-1, or salivary GOAT levels (all *p* > 0.05). *** *p* < 0.001. A significant sex difference was observed.

Serum concentrations of both biomarkers were significantly elevated in the epilepsy group compared to controls. Serum nesfatin-1 levels showed a nearly 5-fold increase (44.04 (IQR 38.19–76.72) vs. 8.65 (IQR 7.82–9.01) ng/mL, *p* < 0.001; Figure 1A). This elevation was observed across both sexes, with women showing median levels of 40.33 (IQR 36.81–49.85) ng/mL and men 56.57 (IQR 40.84–84.42) ng/mL, (*p* = 0.193), compared to HC women 9.04 (IQR 8.80–9.37) ng/mL and men 8.14 (IQR 7.48–8.66) ng/mL.

Serum GOAT levels were 3.5 times higher in patients (4.90 (IQR 4.17–6.66) vs. 1.41 (IQR 1.21–1.79) ng/mL in HCs, *p* < 0.001; Figure 1C). Women had significantly higher GOAT levels than men (5.80 (IQR 4.78–7.73) vs. 4.16 (IQR 3.18–4.90) ng/mL, *p* = 0.019).

In saliva, nesfatin-1 levels were significantly higher in patients than in controls, with a median of 4.58 (IQR 3.06–7.00) vs. 2.02 (IQR 1.45–2.41) ng/mL, *p* < 0.001 (Figure 1B). In contrast, salivary GOAT levels showed a significant 9-fold reduction in the epilepsy group (0.15 (IQR 0.09–0.23) vs. 1.38 (IQR 1.27–1.74) ng/mL, *p* < 0.001, Figure 1D), revealing a divergent pattern between systemic and local oral secretion. No significant sex difference was observed in salivary nesfatin-1 (women: 3.53 (IQR 3.02–4.96) ng/mL vs. men: 5.27 (IQR 4.02–19.56) ng/mL, *p* = 0.151) or salivary GOAT levels (women: 0.13 (IQR 0.05–0.23) ng/mL vs. men: 0.18 (IQR 0.11–0.23) ng/mL, *p* = 0.478).

### 3.3. Correlation Analysis

**Figure 2:**
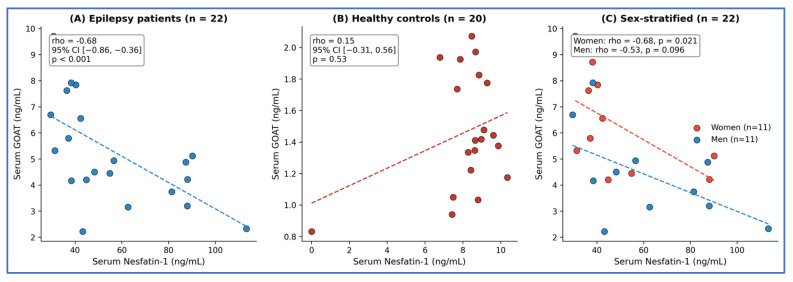
Correlation between serum nesfatin-1 and GOAT levels. (**A**) Epilepsy patients (n = 22) showed a significant inverse correlation (rho = −0.68, 95% CI [−0.86, −0.36], *p* < 0.001). (**B**) Healthy controls (n = 20) showed no significant correlation (rho = 0.15, *p* = 0.53). (**C**) Sex-stratified analysis of epilepsy patients: women (n = 11, red; rho = −0.68, *p* = 0.021) and men (n = 11, blue; rho = −0.53, *p* = 0.096). Dashed lines represent linear trend lines.

Spearman’s correlation analysis revealed a significant inverse relationship between serum nesfatin-1 and GOAT levels in patients with epilepsy (rho = −0.68, 95% CI [−0.86, −0.36], *p* < 0.001; Figure 2A). This inverse relationship was absent in controls (rho = 0.15, 95% CI [−0.31, 0.56], *p* = 0.53; Figure 2B). Partial Spearman correlation controlling for age, sex, and BMI confirmed the independence of this relationship (partial rho = −0.60, *p* = 0.007). Influence diagnostics identified one observation with elevated Cook’s distance (0.266, threshold = 0.182); sensitivity analysis excluding this point strengthened the correlation (rho = −0.72, 95% CI [−0.88, −0.42], *p* < 0.001). Bootstrap resampling (2000 replicates) yielded a median rho of −0.67 (95% CI: [−0.83, −0.37]), confirming the stability of this finding.

Comprehensive pairwise Spearman correlations across all biomarker combinations are presented in Supplementary Table S1. Within the epilepsy group, the serum nesfatin-1 vs. GOAT correlation was the only significant pairwise association; no significant correlations were observed in saliva or across compartments. In controls, the only significant association was between serum GOAT and salivary nesfatin-1 (rho = 0.48, *p* = 0.034). Notably, when all 42 participants were pooled, serum nesfatin-1 and GOAT showed a strong positive correlation (rho = 0.67, *p* < 0.001), reflecting the co-elevation of both biomarkers in patients with epilepsy relative to controls. This reversal—from a positive association at the group level to a negative association within patients—indicates that the inverse relationship is a disease-specific phenomenon. The pooled analysis also revealed a strong negative correlation between serum and salivary GOAT (rho = −0.81, *p* < 0.001), quantitatively confirming the tissue-specific divergence described in Section 3.2.

### 3.4. Influence of Metabolic and Clinical Factors

Biomarker levels were independent of body composition and age. Correlation analysis showed no significant relationship between BMI and serum nesfatin-1 (*p* = 0.325) or serum GOAT (*p* = 0.314) within the patient group.

### 3.5. Influence of Epilepsy Type and Sex

When stratified by epilepsy type, both nesfatin-1 and GOAT levels were similarly elevated in FE (n = 6) and GE (n = 16) patients compared with HCs, with no significant difference between subtypes (nesfatin-1: *p* = 0.449; GOAT: *p* = 0.494).

The inverse correlation between serum nesfatin-1 and GOAT was examined within each epilepsy subtype. In patients with GE (n = 16), the inverse correlation remained statistically significant (rho = −0.67, *p* = 0.006). In patients with FE (n = 6), a strong directional trend was observed (rho = −0.77, *p* = 0.103), but it did not reach statistical significance, likely due to the small subgroup size.

Women exhibited significantly higher serum GOAT levels than men (5.80 (IQR 4.78–7.73) vs. 4.16 (IQR 3.18–4.90) ng/mL, *p* = 0.019). Furthermore, the inverse correlation between nesfatin-1 and GOAT was statistically significant in women (rho = −0.68, 95% CI [−0.91, −0.14], *p* = 0.021), while men demonstrated a similar directional trend that did not reach statistical significance (rho = −0.53, 95% CI [−0.86, 0.11], *p* = 0.096). Given the small subgroup sizes (n = 11 each), these sex-stratified findings should be considered preliminary and require confirmation in larger cohorts (Figure 2C).

Notably, serum nesfatin-1 levels also differed significantly between women and men HCs (9.04 (IQR 8.80–9.37) vs. 8.14 (IQR 7.48–8.66) ng/mL, *p* = 0.010).

## 4. Discussion

### 4.1. Nesfatin-1 Elevation

Our finding of a significantly elevated serum nesfatin-1 level (5-fold increase) aligns with the prevailing literature, which shows that 80% of studies in our systematic review reported increases, particularly in untreated or active epilepsy cases [15]. The magnitude of this elevation matches previous reports by Aydin et al., who noted persistent elevations even after treatment, suggesting that nesfatin-1 may be a stable marker of the epileptic state rather than just a transient post-ictal response [17,18].

Nesfatin-1 elevation was consistent across all types of epilepsy in our cohort, including both focal and generalized forms. This suggests that this biomarker alteration represents a common pathophysiological mechanism in the adolescent brain rather than a syndrome-specific phenomenon. Erkec et al. and Yu et al. also observed consistent elevation regardless of epilepsy type [19,20]. Our cohort included clinically heterogeneous epilepsy syndromes, ranging from idiopathic GE to rarer conditions such as SHE and Landau–Kleffner syndrome. Despite this heterogeneity, biomarker elevations were consistent across subtypes, with no significant differences between focal and generalized forms. This supports the interpretation that the observed alterations reflect a shared seizure-driven neuroendocrine mechanism rather than syndrome-specific pathophysiology. However, the representation of rarer syndromes by single cases limits the ability to draw syndrome-specific conclusions.

The potential mechanisms underlying this elevation likely involve multiple pathways. Nesfatin-1 reduces neuronal excitability by hyperpolarizing dopaminergic neurons in the substantia nigra through potassium channel activation [10]. Experimental evidence demonstrates anticonvulsant effects by reducing oxidative stress and neuroinflammation [21,22]. The peptide may therefore represent an endogenous compensatory mechanism that protects against seizure activity, potentially mediated by modulation of the hypothalamic–pituitary–adrenal axis and stress response pathways [23]. This compensatory role is further contextualized by recent evidence implicating nicotinamide adenine dinucleotide phosphate (NADPH) oxidase 2 (NOX2)-derived reactive oxygen species (ROS) as key drivers of seizure susceptibility and epileptogenesis. Selective NOX2 inhibition has been shown to reduce seizure severity, oxidative DNA damage, and neuroinflammatory cytokine expression in experimental models [24,25]. The documented antioxidant and anti-inflammatory properties of nesfatin-1 may thus represent an endogenous counterbalance to NOX2-mediated oxidative stress. Additionally, the thioredoxin antioxidant system, comprising thioredoxin, thioredoxin reductase (TXNRD), and NADPH, has been implicated in the pathophysiology of epilepsy. Thioredoxin reductase 1 (TXNRD1), the selenoprotein enzyme responsible for reducing oxidized thioredoxin and maintaining cellular redox homeostasis, has been implicated in epilepsy pathophysiology; TXNRD1 mutations are linked to juvenile myoclonic epilepsy, and thioredoxin overexpression attenuates kainate-induced seizure severity in experimental models [26]. NOX2-driven NADPH consumption may deplete the reducing equivalents required by both the thioredoxin and glutathione peroxidase systems, creating a pro-oxidant environment that amplifies seizure-associated neuronal injury. The elevation of nesfatin-1 may therefore reflect a broader antioxidant defense response activated by redox imbalance in epilepsy [27].

### 4.2. GOAT Elevation

Our observation of elevated serum GOAT contributes essential data to the heterogeneous literature. The elevation of serum GOAT likely reflects a compensatory mechanism that increases the production of acylated (active) ghrelin, which has known neuroprotective and anti-convulsant properties [15].

The anticonvulsant effects of GOAT observed in experimental models suggest that our patient cohort may represent an endogenous compensatory response [13,28]. Ghrelin acts through the growth hormone secretagogue receptor 1a (GHSR1a), which is found throughout the brain, especially in areas prone to seizures, such as the hippocampus and cortex. There, it may modulate neuronal excitability and provide neuroprotection [11,13,14]. GOAT catalyzes the acylation of ghrelin, which is required for ghrelin to bind and activate GHSR1a; thus, GOAT levels directly determine the proportion of biologically active (acylated) ghrelin available for receptor signaling [12]. Marchiò et al. found that higher ghrelin levels were associated with a better response to ASMs [29]. This corroborates our hypothesis and corresponds with our finding that patients exhibiting elevated levels were seizure-active yet not classified as drug-resistant.

### 4.3. Inverse Correlation Between Nesfatin-1 and GOAT

The most significant finding of our study is the notable inverse correlation between serum nesfatin-1 and GOAT levels in patients with epilepsy, which was not observed in HCs. This inverse relationship is noteworthy given nesfatin-1 and ghrelin’s opposite effects in appetite regulation and energy homeostasis [30,31,32]. Nesfatin-1 functions as an anorexigenic peptide that suppresses food intake and interacts with melanocortin signaling pathways [8], while ghrelin is a potent orexigenic hormone that stimulates appetite via neuropeptide Y and agouti-related peptide pathways [30]. However, both peptides modulate neuronal excitability, suggesting that their coordinated dysregulation in epilepsy extends beyond metabolic functions [10].

The mechanistic basis for this inverse correlation warrants investigation. One possibility is that both peptides respond to common upstream regulatory factors altered in epilepsy, such as inflammatory mediators, oxidative stress, or disrupted neurotransmitter systems [22,33,34,35,36]. Nesfatin-1 and ghrelin may also regulate each other’s expression or secretion, directly or indirectly, through pathways that are yet unknown [37]. The observation that this inverse correlation was present in serum but absent in saliva points to tissue-specific regulation and suggests that the relationship reflects CNS or systemic changes rather than a local salivary gland physiology [18,38].

The specificity of this inverse correlation to epilepsy is further underscored by the pooled analysis (n = 42), which revealed a strong positive correlation between serum nesfatin-1 and GOAT (rho = 0.67, *p* < 0.001). This positive association at the group level reflects the co-elevation of both biomarkers in patients relative to controls. The reversal from a positive pooled correlation to a negative within-patient correlation indicates that the inverse relationship emerges only in the context of epilepsy-related pathophysiology, supporting our hypothesis that these neuropeptides undergo coordinated but opposing dysregulation specific to the disease state.

The sex-specific analysis showed a significant inverse correlation in women (rho = −0.68, *p* = 0.021) and a similar, though less pronounced, trend in men (rho = −0.53, *p* = 0.096). Given the small subgroup sizes (n = 11 each), these findings are preliminary and require confirmation in adequately powered, sex-stratified studies.

### 4.4. Tissue-Specific Regulation: The Saliva–Serum Divergence in GOAT

We observed a significant divergence in GOAT levels between serum (elevated) and saliva (reduced 9-fold). Previous research has demonstrated that salivary glands can locally synthesize both ghrelin and nesfatin-1 [18,38]. The different pattern we observed suggests that local control of salivary glands differs significantly from systemic and CNS control. This may indicate modified autonomic nervous system regulation of salivary glands in epilepsy [39], variations in the local inflammatory or metabolic environment, or differential expression of ghrelin-processing enzymes, such as GOAT, among tissues [40,41].

The comprehensive correlation analysis further supports this tissue-specific regulation: serum and salivary GOAT showed a strong negative correlation in the pooled sample (rho = −0.81, *p* < 0.001), quantitatively confirming the opposing directionality between compartments. Within patients, this cross-compartment correlation was weaker and non-significant (rho = −0.25, *p* = 0.26), suggesting that the divergent pattern is most apparent when comparing the disease state to the healthy baseline.

### 4.5. Sex-Based Regulation of the GOAT–Nesfatin Axis

Our data showed that female patients had significantly higher GOAT levels and a more robust inverse correlation with nesfatin-1 than men. The observation that nesfatin-1 levels also differed significantly between women and men HC (*p* = 0.010) further supports the notion that sex hormones play a role in baseline nesfatin-1 regulation, which may be amplified in the epileptic state [42]. This aligns with neuroendocrine models, in which estrogen and other sex hormones act as potent modulators of the ghrelin-GOAT system [43]. In adolescent populations, this sex-specific signaling may be particularly sensitive. The fact that the correlation was significant only in women suggests that the “nesfatin-GOAT axis” might be a more reliable biomarker in women, or that the pathomechanism of seizure-induced metabolic stress differs by sex [42,44].

### 4.6. Influence of Anti-Seizure Medications

All patients in our cohort were receiving ASMs at the time of sampling, which may have influenced neuropeptide levels. Valproic acid has been associated with altered ghrelin levels and weight gain in pediatric epilepsy populations [45,46], while carbamazepine may reduce ghrelin concentrations [47]. Topiramate has been reported to decrease both body weight and appetite-related hormones [48,49]. However, Aydin et al. observed that nesfatin-1 elevations persisted even after treatment initiation, suggesting these elevations may reflect the epileptic state itself rather than medication effects [17,18]. Similarly, Marchiò et al. reported that higher ghrelin levels were associated with positive ASM response, indicating a complex bidirectional relationship between ASMs and the ghrelin-GOAT system [29]. Our study was not designed to disentangle individual ASM effects, as patients received various medications including valproate, levetiracetam, lamotrigine, and oxcarbazepine. Future studies should systematically assess ASM type, duration of treatment, polytherapy status, and ideally include drug-naive patients at diagnosis to isolate disease-specific from treatment-related neuropeptide alterations.

### 4.7. Independence from BMI and Clinical Subtypes

A critical strength of our study is demonstrating that biomarker alterations occur independent of metabolic factors. BMI values were comparable between groups, and neither peptide correlated with BMI in epilepsy patients. This is important given the established roles of both neuropeptides in obesity and metabolic regulation [9,50].

Previous studies have shown conflicting results regarding the relationship between these peptides and body weight in epilepsy, particularly in the context of valproic acid-associated weight gain [45,46,51]. Our findings suggest that in adolescent epilepsy patients without significant obesity, the alterations in nesfatin-1 and GOAT reflect epilepsy-specific pathophysiology rather than secondary metabolic changes [48,49].

### 4.8. Clinical Implications

Our findings suggest several potential clinical applications that require further investigation: (1) The marked elevations may support epilepsy diagnosis as part of a multi-biomarker panel, particularly when clinical or electroencephalogram (EEG) findings are ambiguous; (2) The correlation of nesfatin-1 with seizure frequency reported in previous studies [20,52,53] suggests these biomarkers may track disease activity; (3) Higher ghrelin levels associated with positive ASM response [29,54] raise the possibility of using these biomarkers to predict treatment efficacy; (4) The inverse correlation provides a novel window into epilepsy pathophysiology, potentially revealing new therapeutic targets.

Our neuropeptide biomarker findings should be considered within the broader epilepsy biomarker landscape, which encompasses complementary domains, including immune-mediated biomarkers, such as autoantibodies to neuronal surface proteins (N-methyl-D-aspartate receptor (NMDAR), leucine-rich glioma-inactivated-1 protein (LGI1), contactin-associated protein-like 2 (CASPR2), GABA receptors) [55], and neuronal injury markers, such as neurofilament light chain and tau [55]. While these approaches primarily reflect autoimmune or neurodegenerative processes, the nesfatin-1/GOAT axis offers a distinct window into metabolic-excitability coupling that may complement existing biomarker panels in a multi-modal diagnostic framework.

It is also worth considering whether the observed nesfatin-1/GOAT dysregulation is specific to epilepsy or represents a shared neuroendocrine signature across neuropsychiatric conditions. Both nesfatin-1 and ghrelin have been independently reported to be altered in depression and anxiety disorders, with nesfatin-1 elevated in major depressive disorder [56] and ghrelin dysregulated in stress-related conditions [57]. Whether the specific inverse correlation pattern identified here is unique to epilepsy or present to a lesser degree in comorbid psychiatric conditions warrants investigation in future studies using matched patient populations.

However, clinical implementation requires establishing standardized measurement protocols, defining age-specific reference ranges, and prospectively validating biomarker utility in larger multicenter cohorts. Cost-effectiveness analyses will also be needed to justify routine clinical use.

### 4.9. Limitations

Several limitations should be acknowledged. First, the cross-sectional design prevents assessing temporal changes in biomarker levels in relation to seizure frequency or treatment response. Longitudinal studies are needed to determine whether these biomarkers track with disease progression and treatment efficacy over time. The relatively small numbers in epilepsy subgroups (focal n = 6, generalized n = 16) limit our power to detect syndrome-specific effects.

We lacked detailed seizure frequency data immediately preceding sampling, which might have influenced acute biomarker levels. Previous studies have shown that nesfatin-1 levels decrease acutely after seizures [17]. We measured GOAT without distinguishing between acylated and des-acyl ghrelin forms, which have distinct biological activities and may exhibit different epilepsy patterns [58,59]. Additionally, we did not systematically assess pubertal status using Tanner staging. Given the influence of sex hormones on neuropeptide regulation, future studies should include detailed pubertal staging to determine whether biomarker levels vary with sexual maturation status [45,47,60].

Additionally, all patients were receiving anti-seizure medications at the time of sampling. Although the existing literature suggests that neuropeptide elevations persist despite treatment [17,18], we cannot exclude medication-related effects on biomarker levels. The absence of drug-naive patients and the heterogeneity of ASM regimens in our cohort preclude definitive conclusions about disease-specific versus treatment-related alterations. Our findings should therefore be considered exploratory and hypothesis-generating, requiring validation in larger, ideally multicenter cohorts that include drug-naive patients and systematic ASM stratification.

Finally, our adolescent population with relatively recent epilepsy onset may not generalize to adult epilepsy, long-standing disease, or specific epilepsy syndromes not represented in our cohort. Validation in diverse epilepsy populations is essential.

### 4.10. Future Directions

Our findings open several avenues. Longitudinal studies tracking neuropeptide levels relative to seizure control, medication changes, and disease progression would establish their utility in monitoring. Investigating mechanisms underlying the inverse correlation could yield pathophysiological insights and therapeutic targets. Examining these biomarkers in specific epilepsy syndromes, particularly drug-resistant epilepsy, may identify subgroups in which biomarker assessment is most useful.

Studies should explore whether interventions modulating nesfatin-1 or ghrelin affect seizure control. Experimental evidence suggests ghrelin has anticonvulsant properties [13], and nesfatin-1 exerts neuroprotective effects [22], raising therapeutic possibilities requiring careful safety and efficacy evaluation.

## 5. Conclusions

This exploratory study demonstrates marked elevation of both the nesfatin-1 and ghrelin-GOAT axis in adolescent epilepsy patients. Comprehensive correlation analysis revealed a significant and robust inverse correlation between serum nesfatin-1 and GOAT, specific to epilepsy; independent of age, sex, and BMI; and absent in both healthy controls and the pooled sample, where a positive association was observed. The tissue-specific regulation observed, with opposite serum and saliva patterns for GOAT, adds complexity to our understanding of neuropeptide dysregulation in epilepsy.

These findings support the potential of nesfatin-1 and GOAT as candidate biomarkers for epilepsy, warranting validation in larger, diverse populations, and provide new insights into the neuroendocrine pathophysiology of epilepsy.

## Figures and Tables

**Table 1:** Demographic and clinical characteristics of study participants. Data are presented as median (IQR). *p*-values were calculated using Mann–Whitney U for continuous variables and chi-square test for sex distribution (*).

Characteristic	Epilepsy (n = 22)	Controls (n = 20)	*p*-Value
Age (years), mean ± SD	13.1 ± 2.0	12.3 ± 2.2	0.230
Age range	11–16	10–17	-
Sex (F/M)	11/11	8/12	0.753 *
BMI (kg/m^2^) Median (IQR)	20.01 (18.66–23.74)	19.82 (18.00–22.01)	0.583
Serum Nesfatin-1 (ng/mL) Median (IQR)	44.04 (38.19–76.72)	8.65 (7.82–9.01)	*p* < 0.001
Salivary Nesfatin-1 (ng/mL) Median (IQR)	4.58 (3.06–7.00)	2.02 (1.45–2.41)	*p* < 0.001
Serum GOAT (ng/mL) Median (IQR)	4.90 (4.17–6.66)	1.41 (1.21–1.79)	*p* < 0.001
Salivary GOAT (ng/mL) Median (IQR)	0.15 (0.09–0.23)	1.38 (1.27–1.74)	*p* < 0.001

## Data Availability

The original contributions presented in this study are included in the article/Supplementary Materials. Further inquiries can be directed to the corresponding author.

## Supplementary Materials

The following supporting information can be downloaded at https://www.mdpi.com/article/10.3390/biom16050658/s1, Table S1: Comprehensive pairwise Spearman correlations across all biomarker combinations.

## Abbreviations

The following abbreviations are used in this manuscript:

ASM	Anti-seizure medication
BMI	Body-mass index
CASPR2	Contactin-associated protein-like 2
CNS	Central nervous system
CV	Coefficient of variation
EEG	Electroencephalogram
ELISA	Enzyme-linked immunosorbent assay
FE	Focal epilepsy
GABA	Gamma-aminobutyric acid
GE	Generalized epilepsy
GHSR1a	Growth hormone secretagogue receptor 1a
GOAT	Ghrelin O-acyltransferase
HCs	Healthy controls
IGE	Idiopathic generalized epilepsy
ILAE	International League Against Epilepsy
IQR	Interquartile range
JME	Juvenile myoclonic epilepsy
LGI1	Leucine-rich glioma-inactivated 1 protein
MBOAT4	Membrane-bound O-acyltransferase domain-containing protein 4
NADPH	Nicotinamide adenine dinucleotide phosphate
NMDAR	N-methyl-D-aspartate receptor
NOX2	NADPH oxidase 2
ROS	Reactive oxygen species
SHE	Sleep-related hypermotor epilepsy
TXNRD	Thioredoxin reductase

## References

[1] Fiest K.M., Sauro K.M., Wiebe S., Patten S.B., Kwon C.S., Dykeman J., Pringsheim T., Lorenzetti D.L., Jetté N. (2017). Prevalence and incidence of epilepsy: A systematic review and meta-analysis of international studies. Neurology.

[2] Kalilani L., Sun X., Pelgrims B., Noack-Rink M., Villanueva V. (2018). The epidemiology of drug-resistant epilepsy: A systematic review and meta-analysis. Epilepsia.

[3] Löscher W., Potschka H., Sisodiya S.M., Vezzani A. (2020). Drug Resistance in Epilepsy: Clinical Impact, Potential Mechanisms, and New Innovative Treatment Options. Pharmacol. Rev..

[4] Mazhit A., Akbay B., Trofimov A., Karapina O., Duysenbi S., Tokay T. (2026). Epileptogenesis and Epilepsy Treatment: Advances in Mechanistic Understanding, Therapeutic Approaches, and Future Perspectives. Int. J. Mol. Sci..

[5] Engel J., Pitkänen A., Loeb J.A., Dudek F.E., Bertram E.H., Cole A.J., Moshé S.L., Wiebe S., Jensen F.E., Mody I. (2013). Epilepsy biomarkers. Epilepsia.

[6] Clynen E., Swijsen A., Raijmakers M., Hoogland G., Rigo J.M. (2014). Neuropeptides as Targets for the Development of Anticonvulsant Drugs. Mol. Neurobiol..

[7] Morita-Sherman M., Trinka E., Kwan P., Ikeda A., Cho M., Hampel H. (2025). Precision medicine for epilepsy: Challenges and perspectives for an optimized clinical care pathway. Front. Neurol..

[8] Oh I.S., Shimizu H., Satoh T., Okada S., Adachi S., Inoue K., Eguchi H., Yamamoto M., Imaki T., Hashimoto K. (2006). Identification of nesfatin-1 as a satiety molecule in the hypothalamus. Nature.

[9] Kojima M., Hosoda H., Date Y., Nakazato M., Matsuo H., Kangawa K. (1999). Ghrelin is a growth-hormone-releasing acylated peptide from stomach. Nature.

[10] Li C., Zhang F., Shi L., Zhang H., Tian Z., Xie J., Jiang H. (2014). Nesfatin-1 Decreases Excitability of Dopaminergic Neurons in the Substantia Nigra. J. Mol. Neurosci..

[11] Zigman J.M., Jones J.E., Lee C.E., Saper C.B., Elmquist J.K. (2006). Expression of ghrelin receptor mRNA in the rat and the mouse brain. J. Comp. Neurol..

[12] Davis T.R., Pierce M.R., Novak S.X., Hougland J.L. (2021). Ghrelin octanoylation by ghrelin O-acyltransferase: Protein acylation impacting metabolic and neuroendocrine signalling. Open Biol..

[13] Portelli J., Michotte Y., Smolders I. (2012). Ghrelin: An emerging new anticonvulsant neuropeptide. Epilepsia.

[14] Banks W.A., Tschöp M., Robinson S.M., Heiman M.L. (2002). Extent and direction of ghrelin transport across the blood-brain barrier is determined by its unique primary structure. J. Pharmacol. Exp. Ther..

[15] Yetkin O., Sojka A., Bartosik Y., Steinborn B., Dorocka-Bobkowska B., Zarowski M. (2025). Exploring the roles of nesfatin-1 and ghrelin as potential biomarkers in human epilepsy. Proceedings of the 36th International Epilepsy Congress.

[16] R Core Team (2025). R: A Language and Environment for Statistical Computing.

[17] Aydin S., Dag E., Ozkan Y., Arslan O., Koc G., Bek S., Kirbas S., Kasikci T., Abasli D., Gokcil Z. (2011). Time-dependent changes in the serum levels of prolactin, nesfatin-1 and ghrelin as a marker of epileptic attacks young male patients. Peptides.

[18] Aydin S., Dag E., Ozkan Y., Erman F., Dagli A.F., Kilic N., Sahin I., Karatas F., Yoldas T., Barim A.O. (2009). Nesfatin-1 and ghrelin levels in serum and saliva of epileptic patients: Hormonal changes can have a major effect on seizure disorders. Mol. Cell. Biochem..

[19] Erkec O.E., Milanlioǧlu A., Komuroglu A.U., Kara M., Huyut Z., Keskin S. (2021). Evaluation of serum ghrelin, nesfatin-1, irisin, and vasoactive intestinal peptide levels in temporal lobe epilepsy patients with and without drug resistance: A cross-sectional study. Rev. Assoc. Med. Bras..

[20] Yu A.H., Liu Q., Sun C.J. (2021). The clinical value of EEG monitoring and silver nanoparticles to detect the levels of serum Nesfatin-1, S100β and neuron-specific enolase in evaluating the severity and prognosis of epilepsy. Mater. Express.

[21] Keloglan S.M., Aycik F.B., Kocacan S.E., Yazgan B., Ayyildiz M., Agar E. (2023). Nesfatin-1 exerts anticonvulsant effect by reducing oxidative stress in experimental epilepsy model. Acta Neurobiol. Exp..

[22] Arabacı Tamer S., Koyuncuoğlu T., Karagöz Köroğlu A., Akakın D., Yüksel M., Yeğen B. (2022). Nesfatin-1 ameliorates oxidative brain damage and memory impairment in rats induced with a single acute epileptic seizure. Life Sci..

[23] Dore R., Levata L., Lehnert H., Schulz C. (2017). Nesfatin-1: Functions and physiology of a novel regulatory peptide. J. Endocrinol..

[24] Augsburger F., Filippova A., Rasti D., Seredenina T., Lam M., Maghzal G., Mahiout Z., Jansen-Dürr P., Knaus U.G., Doroshow J. (2019). Pharmacological characterization of the seven human NOX isoforms and their inhibitors. Redox Biol..

[25] Singh P.K., Maurya S., Saadi A., Zhang T., Lieb A., Shekh-Ahmad T. (2025). Selective inhibition of NOX2 after status epilepticus attenuates epileptogenesis and cognitive impairment: A sex-dependent study. Redox Biol..

[26] Ohmori I., Ouchida M., Shinohara M., Kobayashi K., Ishida S., Mashimo T. (2022). Novel animal model of combined generalized and focal epilepsy. Epilepsia.

[27] Pearson-Smith J.N., Patel M. (2017). Metabolic Dysfunction and Oxidative Stress in Epilepsy. Int. J. Mol. Sci..

[28] Portelli J., Thielemans L., Ver Donck L., Loyens E., Coppens J., Aourz N., Aerssens J., Vermoesen K., Clinckers R., Schallier A. (2012). Inactivation of the constitutively active ghrelin receptor attenuates limbic seizure activity in rodents. Neurotherapeutics.

[29] Marchiò M., Roli L., Giordano C., Caramaschi E., Guerra A., Trenti T., Biagini G. (2018). High plasma levels of ghrelin and des-acyl ghrelin in responders to antiepileptic drugs. Neurology.

[30] Korbonits M., Goldstone A.P., Gueorguiev M., Grossman A.B. (2004). Ghrelin--a hormone with multiple functions. Front. Neuroendocrinol..

[31] Nass R., Farhy L.S., Liu J., Pezzoli S.S., Johnson M.L., Gaylinn B.D., Thorner M.O. (2014). Age-dependent decline in acyl-ghrelin concentrations and reduced association of acyl-ghrelin and growth hormone in healthy older adults. J. Clin. Endocrinol. Metab..

[32] Whatmore A., Hall C., Jones J., Westwood M., Clayton P. (2003). Ghrelin concentrations in healthy children and adolescents. Clin. Endocrinol..

[33] Arslan G., Ayyildiz M., Agar E. (2014). The interaction between ghrelin and cannabinoid systems in penicillin-induced epileptiform activity in rats. Neuropeptides.

[34] Aslan A., Yildirim M., Ayyildiz M., Güven A., Agar E. (2009). The role of nitric oxide in the inhibitory effect of ghrelin against penicillin-induced epileptiform activity in rat. Neuropeptides.

[35] Ataie Z., Babri S., Golzar M.G., Ebrahimi H., Mirzaie F., Mohaddes G. (2013). GABAB receptor blockade prevents antiepileptic action of ghrelin in the rat hippocampus. Adv. Pharm. Bull..

[36] Keloǧlan S., Yazgan B., Şen F.B., Kocacan S.E., Ayyildiz M., Aǧar E. (2019). Effect of Nesfatin-1 on oxidative stress parameters in experimental epilepsy model. Acta Physiol..

[37] Ergul Erkec O., Algul S., Kara M. (2018). Evaluation of ghrelin, nesfatin-1 and irisin levels of serum and brain after acute or chronic pentylenetetrazole administrations in rats using sodium valproate. Neurol. Res..

[38] Dag E., Aydin S., Ozkan Y., Erman F., Dagli A.F., Gurger M. (2010). Alteration in chromogranin A, obestatin and total ghrelin levels of saliva and serum in epilepsy cases. Peptides.

[39] Wannamaker B.B. (1985). Autonomic nervous system and epilepsy. Epilepsia.

[40] Gahete M.D., Córdoba-Chacón J., Salvatori R., Castaño J.P., Kineman R.D., Luque R.M. (2010). Metabolic regulation of ghrelin O-acyl transferase (GOAT) expression in the mouse hypothalamus, pituitary, and stomach. Mol. Cell. Endocrinol..

[41] Sakata I., Park W.M., Walker A.K., Piper P.K., Chuang J.C., Osborne-Lawrence S., Zigman J.M. (2012). Glucose-mediated control of ghrelin release from primary cultures of gastric mucosal cells. Am. J. Physiol. Endocrinol. Metab..

[42] Hofmann T., Elbelt U., Ahnis A., Rose M., Klapp B.F., Stengel A. (2015). Sex-specific regulation of NUCB2/nesfatin-1: Differential implication in anxiety in obese men and women. Psychoneuroendocrinology.

[43] Börchers S., Krieger J.P., Maric I., Carl J., Abraham M., Longo F., Asker M., Richard J.E., Skibicka K.P. (2022). From an Empty Stomach to Anxiolysis: Molecular and Behavioral Assessment of Sex Differences in the Ghrelin Axis of Rats. Front. Endocrinol..

[44] Pate A.T., Schnell A.L., Ennis T.A., Samson W.K., Yosten G.L.C. (2019). Expression and function of nesfatin-1 are altered by stage of the estrous cycle. Am. J. Physiol. Regul. Integr. Comp. Physiol..

[45] Gungor S., Yücel G., Akinci A., Tabel Y., Ozerol I.H., Yologlu S. (2007). The role of ghrelin in weight gain and growth in epileptic children using valproate. J. Child Neurol..

[46] Greco R., Latini G., Chiarelli F., Iannetti P., Verrotti A. (2005). Leptin, ghrelin, and adiponectin in epileptic patients treated with valproic acid. Neurology.

[47] Prodam F., Bellone S., Casara G., De Rienzo F., Grassino E.C., Bonsignori I., Demarchi I., Rapa A., Radetti G., Bona G. (2010). Ghrelin levels are reduced in prepubertal epileptic children under treatment with carbamazepine or valproic acid. Epilepsia.

[48] Okuyaz C., Kursel O., Komur M., Tamer L. (2012). Evaluation of appetite-stimulating hormones in prepubertal children with epilepsy during topiramate treatment. Pediatr. Neurol..

[49] Ozcelik A.A., Serdaroglu A., Bided A., Arhan E., Soysal S., Demir E., Gucuyener K. (2014). The Effect of Topiramate on Body Weight and Ghrelin, Leptin, and Neuropeptide-Y Levels of Prepubertal Children with Epilepsy. Pediatr. Neurol..

[50] Perello M., Dickson S. (2015). Ghrelin signalling on food reward: A salient link between the gut and the mesolimbic system. J. Neuroendocrinol..

[51] Hasaneen B., Salem N.A., El Sallab S., Elgaml D., Elhelaly R. (2016). Body weight, body composition, and serum ghrelin in epileptic children receiving levetiracetam monotherapy. Egypt. Pediatr. Assoc. Gaz..

[52] Chen M., Xie M., Wan J. (2019). Dynamic variety of serum Nesfatin-1 and its clinical values in evaluation on illness condition and short-term prognosis in patients with epileptic seizure. J. Jilin Univ. Med. Ed..

[53] Shimizu H., Oh-I S., Okada S., Mori M. (2009). Nesfatin-1: An overview and future clinical application. Endocr. J..

[54] Mohamed W.S., Nageeb R.S., Elsaid H.H. (2019). Serum and urine ghrelin in adult epileptic patients. Egypt. J. Neurol. Psychiatry Neurosurg..

[55] Nass R.D., Akgün K., Dague K.O., Elger C.E., Reichmann H., Ziemssen T., Surges R. (2021). CSF and Serum Biomarkers of Cerebral Damage in Autoimmune Epilepsy. Front. Neurol..

[56] Ari M., Ozturk O.H., Bez Y., Oktar S., Erduran D. (2011). High plasma nesfatin-1 level in patients with major depressive disorder. Prog. Neuropsychopharmacol. Biol. Psychiatry.

[57] Lutter M., Sakata I., Osborne-Lawrence S., Rovinsky S.A., Anderson J.G., Jung S., Birnbaum S., Yanagisawa M., Elmquist J.K., Nestler E.J. (2008). The orexigenic hormone ghrelin defends against depressive symptoms of chronic stress. Nat. Neurosci..

[58] Varrasi C., Strigaro G., Sola M., Falletta L., Moia S., Prodam F., Cantello R. (2014). Interictal ghrelin levels in adult patients with epilepsy. Seizure.

[59] Costa A.M., Lo Barco T., Spezia E., Conti V., Roli L., Marini L., Minghetti S., Caramaschi E., Pietrangelo L., Pecoraro L. (2022). Prospective Evaluation of Ghrelin and Des-Acyl Ghrelin Plasma Levels in Children with Newly Diagnosed Epilepsy: Evidence for Reduced Ghrelin-to-Des-Acyl Ghrelin Ratio in Generalized Epilepsies. J. Pers. Med..

[60] Cansu A., Yesilkaya E., Serdaroglu A., Camurdan O., Hirfanoglu T.L., Karaoglu A., Bideci A., Cinaz P. (2012). The Effects of oxcarbazepine and valproate therapies on growth in children with epilepsy. Endocr. Res..

