# Supplementary material for: Inverse Correlation Between Nesfatin-1 and Ghrelin O-Acyltransferase (GOAT) in Adolescents with Epilepsy: A Cross-Sectional Study
Source: Biomolecules. 2026 Apr 28;16(5):658. doi: 10.3390/biom16050658 (PMC13204653; doi:10.3390/biom16050658)
Supplement: Supplementary file 1 [file biomolecules-16-00658-s001.zip › biomolecules-4213404-supplementary.pdf]

Table S1. Comprehensive pairwise Spearman correlations across all biomarker combinations.

Correlations were computed separately for epilepsy patients (n = 22), healthy controls (n = 20), and the pooled sample (n = 42). GOAT = ghrelin O-acyltransferase; CI = confidence interval; n=number, ns = not significant; * $p < 0.05$; *** $p < 0.001$.

Group	Biomarker Pair	n	rho	95% CI	p-Value	Significance
Epilepsy	Serum Nesfatin-1 vs. Serum GOAT	22	−0.677	[−0.855, −0.357]	0.001	***
Epilepsy	Salivary Nesfatin-1 vs. Salivary GOAT	22	−0.027	[−0.443, 0.400]	0.907	ns
Epilepsy	Serum Nesfatin-1 vs. Salivary Nesfatin-1	22	0.025	[−0.400, 0.442]	0.911	ns
Epilepsy	Serum GOAT vs. Salivary GOAT	22	−0.251	[−0.608, 0.191]	0.259	ns
Epilepsy	Serum Nesfatin-1 vs. Salivary GOAT	22	0.359	[−0.074, 0.678]	0.101	ns
Epilepsy	Serum GOAT vs. Salivary Nesfatin-1	22	−0.149	[−0.537, 0.291]	0.510	ns
Controls	Serum Nesfatin-1 vs. Serum GOAT	20	0.149	[−0.314, 0.555]	0.531	ns
Controls	Salivary Nesfatin-1 vs. Salivary GOAT	20	−0.145	[−0.552, 0.318]	0.541	ns
Controls	Serum Nesfatin-1 vs. Salivary Nesfatin-1	20	0.035	[−0.414, 0.470]	0.885	ns
Controls	Serum GOAT vs. Salivary GOAT	20	−0.220	[−0.604, 0.247]	0.352	ns
Controls	Serum Nesfatin-1 vs. Salivary GOAT	20	0.011	[−0.433, 0.452]	0.962	ns

Group	Biomarker Pair	n	rho	95% CI	p-Value	Significance
Controls	Serum GOAT vs. Salivary Nesfatin-1	20	0.477	[0.043, 0.759]	0.034	*
Pooled (n = 42)	Serum Nesfatin-1 vs. Serum GOAT	42	0.668	[0.456, 0.808]	< 0.001	***
Pooled (n = 42)	Salivary Nesfatin-1 vs. Salivary GOAT	42	−0.625	[−0.781, −0.397]	< 0.001	***
Pooled (n = 42)	Serum Nesfatin-1 vs. Salivary Nesfatin-1	42	0.564	[0.314, 0.741]	< 0.001	***
Pooled (n = 42)	Serum GOAT vs. Salivary GOAT	42	−0.808	[−0.893, −0.669]	< 0.001	***
Pooled (n = 42)	Serum Nesfatin-1 vs. Salivary GOAT	42	−0.696	[−0.825, −0.497]	< 0.001	***
Pooled (n = 42)	Serum GOAT vs. Salivary Nesfatin-1	42	0.628	[0.400, 0.782]	< 0.001	***
